# Antibodies against polysaccharide type 3 and pneumococcal proteins demonstrate synergistic protective effect in a highly virulent type 3 invasive disease model in mice

**DOI:** 10.3389/fimmu.2025.1707686

**Published:** 2025-12-12

**Authors:** Taylor C. Stevenson, Heidi Burke, Sara E. Roggensack, Anthony Pratt, Aquib Hossain, Pallab Ghosh, Gilles Besin, Yingjie Lu, Fan Zhang, Richard Malley, Shite Sebastian

**Affiliations:** 1 GSK, Cambridge, MA, United States; 2 Affinivax, Inc., Cambridge, MA, United States; 3 Division of Infectious Diseases, Department of Medicine, Boston Children’s Hospital, Boston, MA, United States

**Keywords:** *Streptococcus pneumoniae* (pneumococcus), serotype 3, vaccine, MAPS, pneumolysin

## Abstract

*Streptococcus pneumoniae* serotype-3 (ST-3) continues to be a major contributor to pneumococcal disease burden despite its inclusion in multivalent pneumococcal conjugate vaccines (PCV). Therefore, there is a pressing need to develop vaccines that can elicit more versatile immune responses against ST-3. To this purpose, our approach was to evaluate MAPS technology-based vaccine candidates in which biotinylated pneumococcal polysaccharides are non-covalently complexed with biotin-binding proteins. One such protein was SPP2, a fusion protein containing a nonhemolytic mutant of pneumolysin (Ply). The immunogenicity of SPP2 as a component of a vaccine candidate containing over 30 pneumococcal capsular polysaccharides, including ST-3 capsule (CPS3), was evaluated in rabbits. The vaccine demonstrated strong immunogenic properties, producing high titers of Ply-neutralizing antibodies and a robust opsonophagocytic antibody response to ST-3. A highly lethal ST-3 pneumococcal invasive disease model, in which antibodies to CPS3 alone are not highly protective, was developed to evaluate whether the inclusion of SPP2 can provide synergistic protection. Active immunization of mice with SPP2- and CPS3-containing vaccine and passive immunization of mice with antisera containing anti-CPS3 and anti-SPP2 antibodies conferred significant protection against death, whereas immunization against either antigen alone did not confer protection, suggesting a synergistic interaction between the protein- and polysaccharide-directed antibodies. These findings strongly support a vaccine approach that includes both pneumococcal polysaccharides and highly conserved disease-specific proteins to overcome the clinical resistance of ST-3 pneumococcal disease to traditional PCVs.

## Introduction

Pneumococcal disease, caused by the Gram-positive bacterium *Streptococcus pneumoniae*, remains a significant public health concern. Most clinically relevant strains of *S. pneumoniae* possess a polysaccharide capsule, of which at least 108 distinct serotypes (STs) have been identified (1–8). However, a limited number of these serotypes are responsible for the majority of pneumococcal diseases worldwide (2, 9–11). Despite the implementation of pneumococcal vaccination programs in children, older adults and individuals with certain underlying medical conditions, pneumococcal disease persists as a major cause of pneumonia, bacteremia, meningitis, sinusitis and otitis media (12). In 2019 alone, over 500,000 deaths could be attributed to *S. pneumoniae*, representing the highest burden of years of life lost (13).

Pneumococcal conjugate vaccines (PCVs), which contain capsular polysaccharides covalently linked to a protein carrier, are the cornerstone of pneumococcal vaccination. Different PCV formulations have been introduced since the first 7-valent (PCV7) vaccine was licensed in 2000. Post-licensure and epidemiological studies have generally confirmed the effectiveness of these vaccines against included serotypes. However, serotype 3 (ST-3), included in the 13-valent vaccine (PCV13) since 2010 remains an exception. Indeed, the incidence of ST-3-attributable disease has remained largely unchanged and continues to contribute significantly to the disease burden in both pediatric and adult populations (14–19).

Although the carriage rates of ST-3 are only modest, a recent study across ten European countries identified ST-3 pneumococcus as the most frequent cause of invasive pneumococcal disease (IPD) in children under 5 years of age, representing 9% of cases. In the same study, 13% of IPD cases were attributable to ST-3 in older adults (20). The failure to control ST-3 is far from inconsequential, as disease outcomes attributable to ST-3 have increased case-fatality rates of approximately 30% following invasive disease, with even higher rates in individuals with identified comorbidities (21–23). Despite the lack of conclusive evidence of vaccine effectiveness against ST-3, recently licensed vaccines (PCV-15, PCV-20, PCV-21) continue to include this important serotype. However, it remains to be seen whether these PCVs with increased valency will have better success against this challenging target.

Several hypotheses have been proposed to explain the poor effectiveness of existing vaccines against ST-3, including the lower immunogenicity of ST-3 capsule (CPS3) (24, 25), the amount of CPS3 produced by most type 3 strains (26), the unique mechanism by which CPS3 is synthesized by the pneumococcal cell (27), or the emergence of a new clade that may be more resistant to the effect of PCV13 (28, 29). In contrast to most of the over 100 STs currently identified, where the capsule is attached to the cell wall via a covalent linkage, only the capsules of ST-3 and ST-37 are membrane-bound to phosphatidylglycerol through a synthase-dependent process, resulting in a non-covalent linkage of the PS to the cell wall. This results in an atypical “mucoid” phenotype, with the bacteria being completely covered by a thick/dense capsular polysaccharide that can be shed during bacterial growth (27). Antibody-mediated killing of bacteria, as measured by an opsonophagocytic assay (OPA), can be adversely impacted by the thickness and/or density of the capsule (30), as demonstrated by the higher correlate of protection threshold calculated for ST-3 (31). Additionally, considerable shedding of this capsule may in turn reduce antibody-mediated protection through: 1) the release of antibody-bound capsules from the bacterial surface; and 2) the potential adsorption of anti-capsular antibodies by shed capsular material (27).

While the underlying mechanisms for ST-3 evasion of PCV vaccination remain unclear, alternative immunological approaches to control type 3 disease may be necessary. Over the past decades, several investigators have explored the possibility of using genetically conserved pneumococcal proteins to target pneumococci in a serotype-transcending manner. These efforts have generally focused on killed whole-cell vaccines, surface-exposed proteins (such as members of the choline-binding protein family or lipoproteins) or the secreted toxin pneumolysin (Ply). While many candidates showed promise in animal models, clinical trials have ultimately been far less encouraging, with no candidate proceeding beyond Phase 2 to date. A strategy that has never been clinically tested is the concurrent use of pneumococcal polysaccharide and conserved *S. pneumoniae* protein. In the case of ST-3, such an approach may be particularly advantageous if indeed the mechanism of resistance to the vaccine is related to a complex interplay between capsule thickness and capsular material shedding.

**Figure 1:**
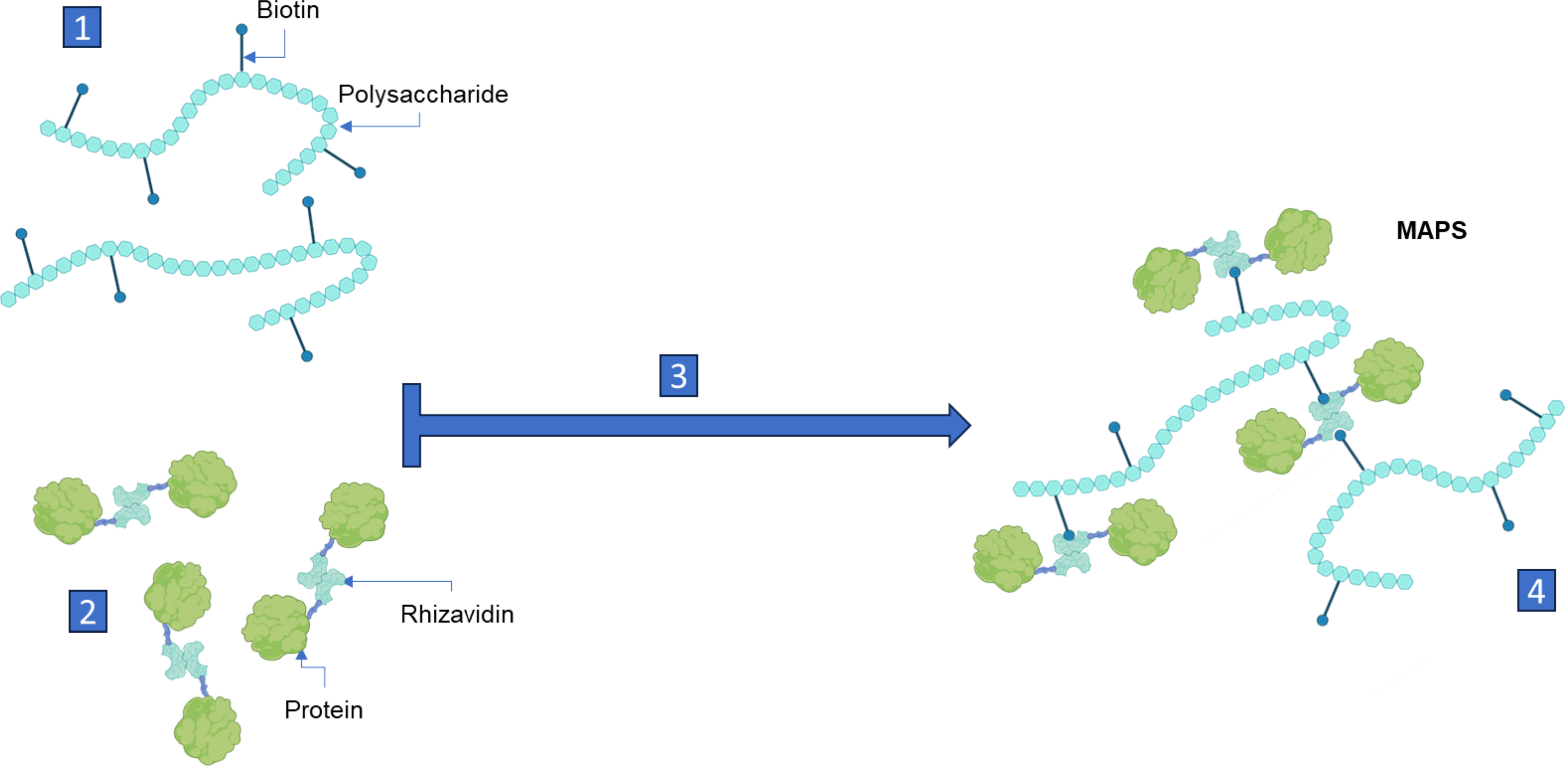
MAPS assembly. 1. Polysaccharide is produced and purified from the relevant pathogen and biotinylated, which provides a scaffolding matrix ​for the final complex. 2. Fusion proteins, comprised of rhizavidin (biotin-binding protein as a dimer) and disease-specific protein, are produced through recombinant DNA technology. 3. Biotinylated polysaccharides and rhizavidin-protein fusions are mixed and incubated. 4. Highly stable and specific ​non-covalent bonds between the biotin and rhizavidin – fusion proteins are formed spontaneously generating a glycocomplex (MAPS). Created with BioRender.com.

To evaluate this hypothesis, we used MAPS technology in which proteins and polysaccharides from a target pathogen are combined into a highly stable, non-covalent glycocomplex. Polysaccharides of interest are first biotinylated and then mixed with proteins of interest that have been genetically fused to the biotin-binding protein rhizavidin (32). Due to the extremely high affinity of rhizavidin for biotin, the non-covalent linkages of the generated glycocomplexes (see **Figure 1**) are highly stable and virtually irreversible (33). When the complexes are used for immunization, they generate robust antibody responses to both polysaccharide and protein components, which has led to the use of MAPS technology across a series of disease targets, as described recently (34). Particularly, Pn-MAPS24v, a pneumococcal vaccine containing 24 capsular polysaccharides complexed with a biotin-binding fusion protein (CP1) that includes two conserved pneumococcal proteins (SP1500 and SP0785) was found to be well tolerated, with an acceptable safety profile, and immunogenic in Phase 1 and 2 clinical trials. It generated robust antibody responses in both adults and toddlers against the protein and polysaccharide components, including CPS3 (35, 36).

In the present study, we evaluate in preclinical settings whether a vaccine containing a combination of type 3 polysaccharide and a fusion protein containing a toxoid of Ply (SPP2) might offer significantly higher protection than either component alone. To this aim, we generated a vaccine candidate containing over 30 pneumococcal capsular polysaccharides and fusion proteins CP1 and SPP2 in the MAPS platform (Pn-MAPS30plus) and evaluated its protective capacity in a lethal mouse model of type 3 pneumococcal invasive disease under experimental conditions in which anti-CPS3 or anti-SPP2 antibodies alone are insufficient for complete protection.

## Materials and methods

### Expression and purification of Ply-toxoid fusion proteins

The PdF construct, which encodes Ply with the three PdT mutations (W433F, D385N, and C428G) (Berry et al., 1995) and a fourth, G294P mutation (37), was codon-optimized for expression in *Escherichia coli* and synthesized by Genscript (Piscataway, NJ) with an N-terminal six-histidine tag followed by a TEV protease cleavage site. The gene sequences encoding the rhizavidin fragment (aa 45–179), CP2a (rhizavidin-SP0435(aa 62-186)-PdT), SPP2 (rhizavidin-PdF-0435(aa 62-186)), each with a six-histidine C-terminal tag (-His), was codon-optimized for *E. coli* expression and synthesized by Genscript. Each synthesized DNA fragment was cloned into pET21b using *Nde*I and *Xho*I restriction sites for rhizavidin-His and *Nde*I and *Blp*I for CP2a-His and His-TEV-PdF. SPP2-His was cloned into pET21b using the CloneEZ PCR cloning kit (Genscript). Two-point mutations were introduced into CP2a-His to replace the Gly 294 codon with Pro, yielding CP2a(G294P)-His using the QuikChange II Site-Directed Mutagenesis Kit (Agilent). The CP1 gene (rhizavidin-1500-0785) DNA sequence was cloned into the pET24a(+) vector using *Nde*I and *Xho*I restriction sites and stop codons (TGA and TAA) were inserted at the 3’-end of the CP1 and CP2a genes.

The plasmid pET21b::His-TEV-PdF was transformed into BL21 cells while pET21b::rhizavidin-His, pET21b::CP2a-His, pET21b::CP2a(G294P)-His, and pET21b::SPP2-His plasmids were all transformed into the origami B (DE3) cell line by heat shock, selecting with 100 µg/mL carbenicillin. The pET24a::CP1 plasmid was transformed into Shuffle T7 Express *E. coli* cells (NEB) by heat shock, selecting with 50 µg/mL kanamycin. Single colonies of transformed cells were grown and induced to test expression and Plasmid DNA was isolated and sequenced to confirm the correct insertion by Genewiz (Cambridge, MA) or Genscript. Expression strains of the his-tagged constructs were grown in LB at 37 °C with 100 µg/mL ampicillin and 30 °C with 50 µg/mL kanamycin for origami B/BL21 and Shuffle T7 express, respectively, shaking at 250 rpm. Cultures were expanded and grown overnight in the same conditions. Overnight cultures were expanded and grown to an OD_600_ = 1–2 prior to induction with 1 mM isopropyl-ß-D-1-thiogalactopyranoside at 16 °C overnight. Cell pellets were harvested by centrifugation, frozen, and lysed by homogenization at 15,000 to 17,000 psi for two cycles in lysis buffer (0.8 M urea, 20 mM Tris pH-8.0, 500 mM NaCl, 10 mM imidazole, 2% Tween-80, 2X HALT protease inhibitor cocktail [Thermo-Fisher], 10 mM MgCl_2_). Cellular debris was pelleted and filtered away from the soluble lysate using a 0.45-µm PES sterile bottle-top filter (Nalgene). Recombinant his-tagged proteins were isolated from the cellular supernatant by nickel affinity chromatography (Complete His-tag purification column, Roche) with binding buffer (0.8 M urea, 20 mM Tris pH-8.0, 500 mM NaCl, 10 mM imidazole), raising imidazole to 250 mM to elute. The His-TEV-PdF protein was cleaved with TEV protease (ProTEV, Promega), then run over nickel affinity column again. The eluents of the Ni-NTA column for all proteins were further purified by anion-exchange chromatography and/or size-exclusion chromatography (SEC; Superdex 200, GE Healthcare) with Tris buffer (20 mM Tris pH-8.0, 150 mM NaCl, 5 mM EDTA). Protein samples at each stage of purification were evaluated using sodium-dodecyl-sulphate polyacrylamide gel electrophoresis (SDS-PAGE) with biotin-4-fluorescein as previously described (38).

### Expression and purification of CP1

Expression and purification of a His-tagged CP1 has been previously described (39). A version of CP1 without a His-tag was used for this work. Briefly, CP1 was expressed in a bioreactor and cell lysis was performed by microfluidization followed by clarification. The purity of CP1 was achieved by precipitation of cellular proteins with ammonium sulphate, centrifugation, and filtration. Further purification was performed by several chromatographic steps including hydrophobic interaction chromatography and anion exchange chromatography. Final material was concentrated by diafiltration, followed by filtration and bulk fill. Purified CP1 was stored at –80 °C. All steps were analyzed for CP1 content and purity by SDS-PAGE.

### Polysaccharide production and biotinylation

Lyophilized *S. pneumoniae* polysaccharides (over 30 serotypes including ST-1 and ST-3 sourced from ATCC in Manassas, VA, Walvax in Kunming, China, or SSI Diagnostica in Hillerød, Denmark) were dissolved in 150 mM NaCl or phosphate-buffered saline (PBS), sterile-filtered, and analyzed to determine their concentration and molecular weight using size exclusion chromatography with multi-angle light scattering (SEC-MALS) (40). As needed, excessively large polysaccharides (including ST-3) were mechanically homogenized to reduce their size, and dilute polysaccharides were concentrated via centrifugal filters with 30-kDa cutoff membranes. Prepared polysaccharides were sterile-filtered again, and their concentrations and sizes were determined using SEC-MALS. Individual serotypes were mixed with serotype-specific combinations of sodium chloride, 1-cyano-4-dimethylaminopyridinium tetrafluoroborate (CDAP), amine-PEG3-biotin, and borate buffered to pH 8.5–9.0 before incubation with end-over-end rotation for 30 min–2 h at room temperature. The desired level of biotinylation was achieved by varying the ratio of CDAP to polysaccharide. The reactions were quenched with 2 M glycine for 30 min. Biotinylated polysaccharides were purified using either tangential flow filtration (TFF) or dialysis with a 20- or 30-kDa membrane against 1 or 4 L of PBS, performed three or four times. Biotinylated polysaccharides were sterile-filtered, and their concentration and size were determined using SEC-MALS.

### MAPS complex formation

CP1 and SPP2 were combined with biotinylated CPS1 at a mass/mass ratio of 2.5:1 and 3:1, respectively and CP1 with biotinylated CPS3 at a ratio of 3.45:1, in 20 mM tris pH 8.0, 150 mM NaCl. The mixtures were incubated with end-over-end rotation at room temperature for 1 h and then overnight at 4 °C. The mixtures were run over a Superdex 200 size exclusion column to separate the complexes from any free protein. Samples of peaks were run on SDS-PAGE, boiled and native to confirm the formation of the complexes. The void peak fractions were combined, and the protein and polysaccharide concentrations were measured by bicinchoninic acid (BCA) protein assay kit (Pierce) and anthrone (CPS3) (41) or Uronic Acid Assay (CPS1), respectively.

The remaining complexes were generated at CP1 or SPP2:polysaccharide ratio based on a predetermined ratio for each biotinylated polysaccharide (typically 6:1 to 8:1, but as high as 20:1). The biotin/rhizavidin complexation reaction was incubated with gentle rocking overnight at room temperature. To remove small amounts of uncomplexed protein, a 300-kDa MWCO Slide-A-Lyzer or Float-A-Lyzer dialysis device was used for dialysis against four liters of PBS. The dialysis process was repeated four times at room temperature, with each session lasting at least four hours, under gentle mixing conditions. After dialysis, each individual MAPS drug substance was filtered using a 0.2 µm syringe filter, and samples were taken for analysis to determine their size and percentage of free protein using size-exclusion chromatography coupled with SEC-MALS. Polysaccharide concentration was determined using nephelometry (42).

### Rabbit immmunization

Rabbit immunization was carried out at Cocalico Biologics (Denver, PA, USA). Male New-Zealand White rabbits (Charles River, Wilmington, MA) were immunized intramuscularly (0.5 mL/dose except for PCV13 which received 0.25 mL/dose) two to three times, two weeks apart, alternating right and left thigh with each vaccination. Proteins were formulated in protein formulation buffer (20 mM Tris, pH 8.0, 150 mM NaCl) to a concentration of 0.2 mg/mL protein and adjuvanted with 1.25 mg/mL aluminum hydroxide (Alhydrogel, InvivoGen)). MAPS platform complexes were formulated in complex formulation buffer (20 mM histidine, pH 5.5, 150 mM NaCl, 1 mM NaH_2_PO_4_, 0.02% Polysorbate-80) to a polysaccharide concentration of 2.4 µg/serotype/mL with 1.25 mg/mL aluminum phosphate (Adju-Phos, InvivoGen). PCV13 was obtained commercially. Serum samples were collected from every animal before each dose and two weeks after the final dose. The rabbits were monitored regularly for signs of morbidity, mortality, and clinical symptoms, as well as changes in body temperature, and food and water consumption. Prior to use in any assay or passive immunization, the rabbit sera were heat-inactivated at 56°C for 30 min and filter-sterilized using a 0.2 µm filter.

### Anti-PdF IgG ELISAs

The ELISAs were performed to quantify PdF-specific serum IgG levels in rabbit samples using an approach described by Zollinger and Boslego to compare antigen-specific polyclonal IgG in a direct ELISA to purified total IgG in a sandwich ELISA (43). Briefly, Nunc-Immuno MaxiSorp 96-well plate(s) (ThermoFisher) were coated with 5 μg/mL of rabbit IgG capture antibody (F(ab’)_2_ Fragment-specific goat anti-rabbit IgG, Jackson Laboratory) in the reference standard wells or 2 μg/mL of PdF antigen diluted in Dulbecco’s phosphate-buffered saline (DPBS) in the sample wells and left overnight at 4 °C. The plate(s) were then washed with DPBST (DPBS + 0.05% Tween-20) and blocked with 200 µL of blocking buffer DPBST + 1% bovine serum albumin (BSA) for 1 h at room temperature. After blocking, the plate(s) were washed and 100 μL of diluted purified rabbit or mouse IgG (MP Biomedicals) or diluted sera samples were added to the reference wells or sample wells, respectively, and incubated for 1 h at room temperature. The purified IgG standard ranged from 400 ng/mL to 3.25 ng/mL in twofold serial dilutions while samples were diluted between 1/100 and 1/125,000 in fivefold serial dilutions. The plate(s) were then washed and incubated with 100 µL of secondary antibody (goat anti-rabbit IgG (Fc)- horseradish peroxidase (HRP) or goat anti-mouse IgG (Fc)-HRP, Bio-Rad) diluted 1:200,000 in DPBST. Plate(s) were then washed and 100 µL of TMB substrate (VWR) was added to each well. The plate(s) was developed for 30 min before the reaction was stopped by adding 100 µL of 1 M HCl to each well. The ELISA plate(s) were read at an absorbance of 450 nm on a SpectraMax i3x Plate Reader using Softmax Pro 7.0. The concentrations of PdF-specific IgG in diluted serum samples in the range defined by the standard curve were determined based on a four-parameter logistic (4PL) nonlinear regression model defined by the purified rabbit IgG in the reference wells.

### Anti-CPS IgG electrochemiluminescence assay

Serotype-specific antibodies in vaccinated rabbits were detected by an electroluminescence-based assay (Meso Scale Discovery [MSD]). Briefly, a multi-sera stock with antibodies against all the Pn-MAPS24v serotypes was prepared. The antibody concentration for each serotype in this undiluted standard was defined as 20,000 arbitrary units per mL (a.u./mL). Eleven three-fold serial dilutions were prepared with a standard using assay buffer, consisting of Dulbecco’s Phosphate-buffered saline (DPBST), 1% Casein, and 5 μg/mL pneumococcal cell wall polysaccharide. Quality control (QC) sera and experimental test sera were also diluted 1000-fold and 5000-fold with assay buffer. All diluted sera were incubated at 2–8 °C overnight to reduce background signal. Biotinylated capsular polysaccharides were each bound to a linker that recognizes a specific spot on one of three MSD U-plex 10-spot plates. Three U-plex plates were coated with the linked polysaccharides (eight polysaccharides per plate) overnight and stored at 2—8 °C. After coating, the U-plex plates were blocked with a blocking buffer (PBS + 1% Casein) and incubated with shaking at room temperature. The U-plex plates were then washed with DPBST before diluted sera were added to the plates and incubated with shaking at room temperature for 1 h. Next, the plates were again washed with DPBST, and either secondary anti-rabbit or anti-mouse SULFO-TAG-conjugated antibody was incubated for 1 h with shaking at room temperature. The plates were washed with DPBST and Read Buffer was added onto the plates, which were then immediately read on a Meso QuickPlex SQ 120 (Model No.1300, Meso Scale Diagnostics). The resulting electrochemiluminescence signals of quality control and test sera concentrations were calculated using the standard curves generated by the reference standards of known concentrations. Twenty-four individual reference standard curves were created based on a 4PL nonlinear regression model by the MSD software (Discovery Workbench version 4.0) and used to determine the concentration of each serotype-specific polysaccharide IgGs in quality control and test sera.

### Opsonization, phagocytosis, and killing assay (OPKA)

The assay was based on the well-established protocol described by Nahm and Burton (44). Frozen stocks of *S. pneumoniae* strain AR003 (45) were thawed and resuspended at 2 × 10^5^ CFU/ml in opsonization buffer (OB) (Hank’s buffered saline with 10% heat inactivated fetal bovine serum and 0.1% (w/v) gelatin). To each well of a round-bottom 96-well plate, 20 µl of heat-inactivated (56 °C, 30 min) rabbit serum diluted appropriately in OB or hank’s buffered saline alone were added followed by 10 µl of bacterial suspension in OB. The bacteria and rabbit sera were incubated at room temperature for 30 min with shaking at 650 rpm. Differentiated HL60 cells (ATCC) were washed with OB and resuspended to 1 × 10^7^ cells/ml in OB. To each well, 10 µl of baby rabbit complement (Pel-Freez Biologicals) and 40 µL of HL60 suspension were added (200 to 1, HL60 to bacteria ratio) followed by incubation at 37 °C with 5% CO_2_ and shaking at 650 rpm for 45 min to phagocytize the bacterial cells. Each plate was transferred to ice and incubated for 20 min. Contents of each well were diluted in water 1/5 and 1/25, and each dilution was then plated on 5% blood agar plates. After overnight incubation at 37 °C with 5% CO_2_, the colony-forming units (CFU) were counted for each sample and dilution, discarding data points with colony counts <1 and >250. The percent killing of AR003 at each serum dilution was determined by taking the average CFU of each plating dilution and normalizing it by the average CFU of the no serum control:


$$\left(\%\;killing\;=\;{\left(1-CFU_{sample}/CFU_{no\;serum}\right)}^{*}100\right).$$

### Hemolysis assay

To measure the hemolytic activities of candidate toxoids, a hemolysis assay was conducted to compare the lysis of rabbit red blood cells (RBCs) at varying concentrations of Ply to each of the fusion protein candidates. The RBCs were washed with chilled DPBS *via* centrifugation at 8,000 × *g* prior to dilution to a final concentration of 2% vol/vol in DBPS. Ply (MyBioSource) and fusion protein candidates were diluted in assay buffer (DPBS, 0.1% w/v BSA, 10 mM dithiothreitol) to respective starting concentrations and then serially diluted twofold six or seven times in a 96-well plate. Amount of 100 µl of each diluted protein, assay buffer alone (0% hemolysis control), or assay buffer + 1% Triton X-100 (100% hemolysis control) were transferred to V-bottomed 96-well plate(s). A volume of 50 µl of 2% RBCs was added to each well of the V-bottom 96-well plate(s) and triturated. The plate(s) were sealed and incubated with shaking for 30 min at 37 °C at 350 rpm. The plates were then centrifuged at 1,000 × *g* for 5 min at room temperature before 100 µl of supernatant from the centrifuged V-bottom 96-well plate(s) was transferred to MaxiSorp 96-well flat-bottom plate(s). The absorbance of the MaxiSorp plate(s) was measured at 545 nm SpectraMax i3x to measure the relative amounts of hemoglobin in the supernatant. The % hemolysis at each protein concentration was calculated by interpolating the line defined by the OD_545 nm_ of the 0% and 100% hemolysis controls. The EC50 (concentration of protein required to achieve 50% hemolysis) was calculated by fitting the dilution series of each protein to a 4PL model fit with nonlinear regression (Prism v9.3.1, GraphPad).

### Pneumolysin neutralization assay

Pneumolysin neutralization assays were performed using the *in vitro* hemolysis assay to determine whether antibodies against SPP2 possess the ability to neutralize the hemolytic activity of native Ply. Briefly, 50 µl of a 20 ng/ml Ply solution was incubated with 50 µl of serially diluted rabbit sera in assay buffer in a V-bottom 96-microwell plate for 1 h at 37 °C with shaking at 450 rpm. Following the initial incubation period, 100 µl of 2% RBCs was added to each well of the V-bottom 96-well plate(s) and the hemolysis portion of the assay was conducted as described above. The serum dilution at which 50% of the hemolytic activity of Ply was inhibited (IC50) was calculated using a two-parameter logistic model fit (minimum and maximum neutralization set to 0 and 100%, respectively) with nonlinear regression (Prism). For samples that did not achieve 50% neutralization with the tested dilutions, the lowest dilution tested was recorded. Geometric mean titers (GMTs) were calculated and 95% confidence intervals (CI) were calculated (Prism).

### Mouse active and passive immunization

Mouse experiments were conducted under approval of the IACUC at Mispro Biotech Services (Cambridge, MA, USA). Female C57BL/6 mice (8-week-old, Jackson Laboratory) were immunized subcutaneously with 0.2 mL/dose, three times, two weeks apart. Mice were immunized for the low inoculum dose challenge study with protein or MAPS complexes formulated in 20 mM NaH_2_PO_4_, 150 mM NaCl, 0.01% (w/v) thiomersal, pH 5.8 to a protein concentration of 25—75 µg/mL and adjuvanted with 1.25 mg/mL Adju-Phos. For the high inoculum dose challenge studies, mice were immunized with protein or complexes formulated with complex formulation buffer adjuvanted with 1.25 mg/mL Adju-Phos to a protein concentration of 25—100 µg/mL. In some high challenge dose groups, mice were given glycoconjugate (PCV13) or MAPS (CPS3-CP1) formulated at a concentration of 4.4 µg/mL of ST-3 capsular polysaccharide with the third dose. For passive immunization, naïve mice were dosed interperitoneally with 0.2 mL of heat-inactivated serum from immunized rabbits one day prior to challenge.

### Mouse sepsis challenge

Three weeks following the final active immunization or one day following passive immunization, the mice were challenged with diluted bacteria from frozen stocks of *S. pneumoniae* ST-3 (AR003) via intraperitoneal injection. The dose of the challenge inoculum varied based on the immunization model being tested. The low challenge dose sufficient to achieve > 80% mortality in naïve mice was 3 × 10^4^ CFU/mouse and was used to evaluate whether CP1 or SPP2 can provide some protection against lethal challenge. The high challenge dose required to overcome protection mediated by CPS3 active immunization was determined to be > 6 x 10^6^ CFU/mouse (**Supplementary Figure 1A**) and was used to evaluate active immunizations with combinations of SPP2 and CPS3. Finally, the challenge dose required to overcome protection mediated by anti-CPS3 passive immunization using selected sera was determined to be > 2 × 10^4^ CFU/mouse (**Supplementary Figure 1B**) and was used to demonstrate the synergistic protection mediated by sera containing both anti-SPP2 and anti-CPS3 antibodies. After challenge, the health status of the mice was closely monitored twice daily, and any mouse reaching clinical endpoints of ruffled fur, decreased mobility, hunched appearance, or greater than 20% weight loss observed for two consecutive days, or closed eyelids during gentle stimulation was euthanized. Typically, mice would experience weight loss up to Day 3–4 after which they were either euthanized based on weight loss and clinical symptoms, or they began recovering.

## Results

### SPP2 detoxification and immunogenicity

**Figure 2:**
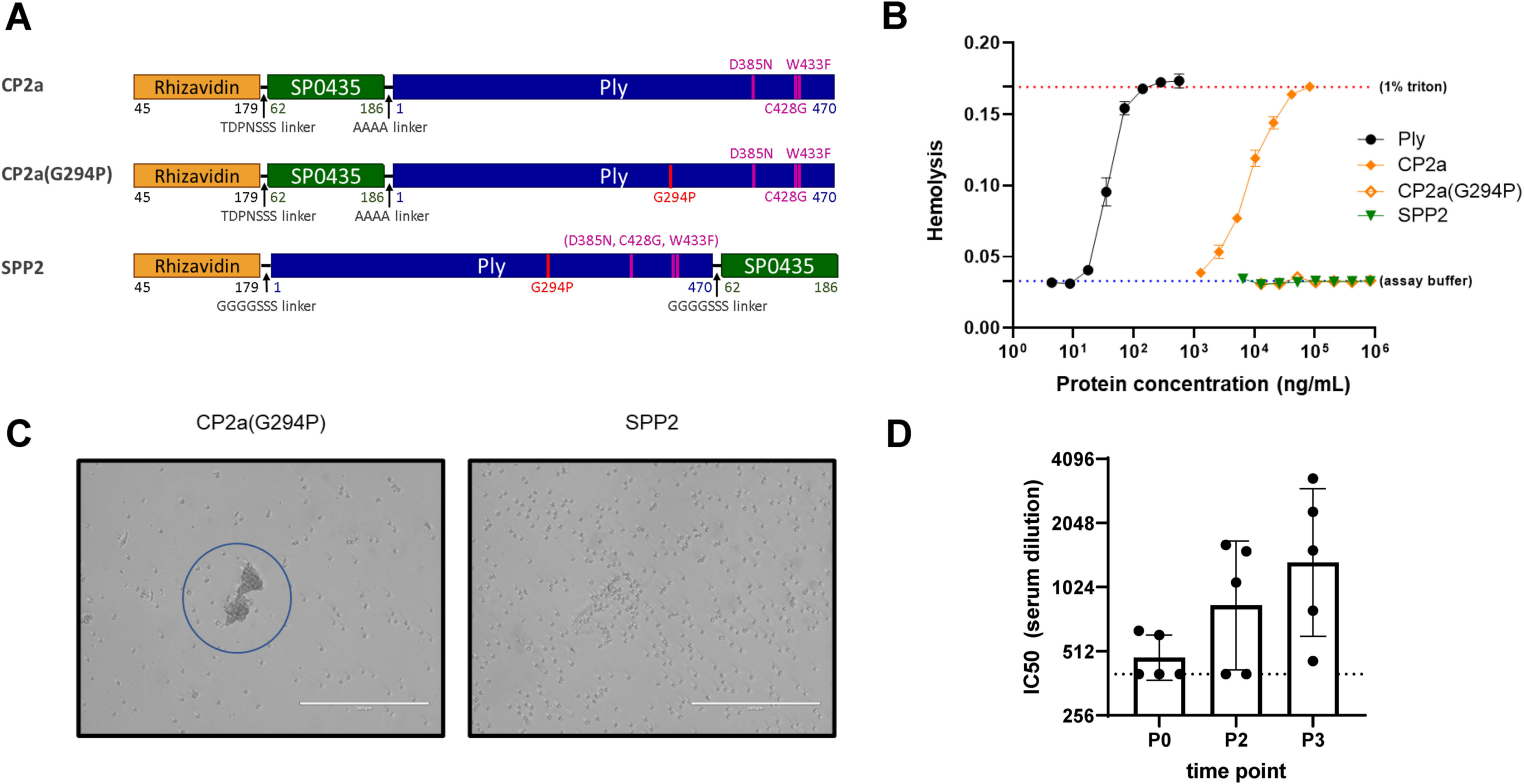
Characteristics of the fusion protein candidates evaluated in this work. **(A)** Schematic representation of the fusion protein candidates. Rhizavidin is the biotin-binding domain that enables formation of glycocomplexes using MAPS technology. SP0435 (elongation factor P) domain contains putative T-cell epitopes to enhance the immunogenicity of the construct. Pneumolysin (Ply) toxoid domains have either three (Ply(D385N, C428G, W433F) or four (Ply(G294P, D385N, C428G, W433F)) amino acid substitutions to abolish hemolytic activity. TDPNSSS, AAAA, or GGGGSSS linkers separate different domains of the fusion proteins as indicated. All constructs were made with 6xHistidine tags to facilitate affinity purification. **(B)** Hemolysis assay comparing lysis of rabbit red blood cells (2% v/v) at different concentrations of Ply, CP2a, CP2a(G294P), and SPP2 to no lysis control (assay buffer) and complete lysis control (1% Triton). Proteins were incubated with rabbit blood cells for 30 min at 37 °C before cells were pelleted via centrifugation. The hemoglobin in the supernatant was evaluated by measuring the absorbance of each sample at 545 nm. Values shown are averages of two technical replicates with error bars representing the range. **(C)** After the hemolysis assay which results are shown in **(B)** the red blood cells were resuspended in the assay plate’s residual liquid and approximately 10 µL were applied to a microscope slide and imaged (20x) with an EVOS microscope. Red blood cells agglutination is visible with CP2a(G294P). **(D)** New Zealand white male rabbits (n=5) were immunized three times with 100 µg each of SPP2 and CP1 adjuvanted with 625 µg of aluminum hydroxide two weeks apart. Sera were collected prior to the first bleed (P0), two weeks post second dose (P2), and two weeks after the final immunization (P3) and heat-inactivated before being evaluated for Ply neutralization. The IC50 is the reported serum dilution of each sample required to neutralize 50% of the Ply activity. The dotted line represents the lowest serum dilution tested (400). P0 and P3 were compared using Dunn’s test and found to be significant (p=0.0362).

Three candidate fusion proteins containing Ply toxoids were evaluated in this work (**Figure 2A**). Three previously described amino acid substitutions in the binding domain of Ply reduced both the hemolytic (C428G, W433F, in combination) and complement activation (D385N) activities of the toxin (46–48). The first candidate fusion protein, CP2a, contains this triple mutant version of Ply C-terminally fused to rhizavidin (residues 45-179) and SP0435 (residues 62-186), the *S. pneumoniae* translation elongation factor P. While CP2a was observed to have greatly reduced hemolytic activity, residual activity required further modification to eliminate it (**Figure 2B**). Single residue changes in the oligomerization domain of Ply have been shown to greatly reduce the hemolytic activity of Ply (37). An additional substitution, Gly to Pro in position 294, was added to the Ply domain of CP2a to create CP2a(G294P). This construct was shown to be even further inactivated when compared with CP2a. However, it displayed a previously unobserved activity causing agglutination of red blood cells (**Figure 2C**), suggesting residual binding activity remained. The N- to C- terminal orientations of SP0435 and the Ply toxoid domains were swapped in the final construct SPP2 so that the binding subdomain of the Ply toxoid was likely shielded by SP0435. SPP2 displayed no hemolytic or hemagglutination activities and was able to generate Ply-neutralizing antibodies in immunized rabbits (**Figure 2D**).

### SPP2 and CPS3 immunization provide synergistic protection

**Figure 3:**
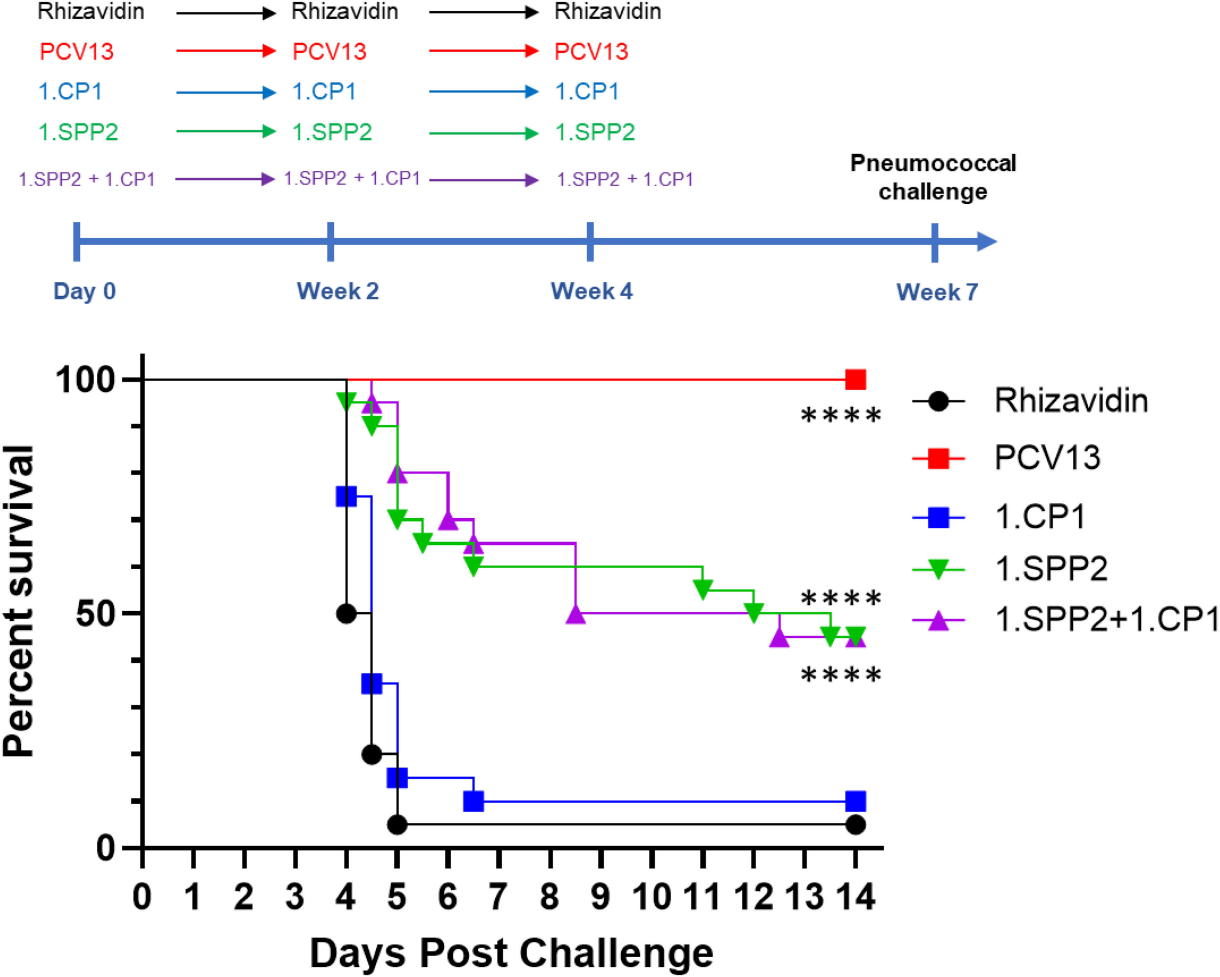
SPP2 vaccination protects against sepsis challenge with ST-3. Mice (n=20 for each group, except n=10 for PCV13 group) were immunized three times, two weeks apart with rhizavidin (5 µg/dose), PCV13 (0.88 µg/PS/dose), CP1 in MAPS on CPS1 (1.CP1,15 µg CP1/dose), SPP2 in MAPS on CPS1 (1.SPP2, 15 µg SPP2/dose), Three weeks after the last immunization, mice were challenged with 2.1 × 10^4^ CFU of S. pneumoniae strain AR003 via intraperitoneal injection. Mice were monitored twice a day and euthanized at clinical endpoints. The percent survival of each group was plotted over two weeks post challenge. Groups immunized with PCV13 or 1.SPP2 showed significant protection (****p<0.0001 with Mantel-Cox test) compared with rhizavidin-immunized control group.

SPP2 was first evaluated for its individual ability to protect against lethal sepsis challenge with a ST-3 strain of *S. pneumoniae* (AR003) in a challenge model under conditions where immunization with PCV13 containing CPS3 is completely protective. **Figure 3** shows that mice are completely protected by three injections of PCV13 at a vaccine dose of 0.88 µg/PS and are significantly protected by three injections of SPP2 complexed with a type 1 capsular polysaccharide that is not relevant to the challenge model (1.SPP2) at a dose of 15 µg of protein in comparison with rhizavidin or 1.CP1 (CP1, complexed with ST-1)-immunized animals (p<0.001, Mantel-Cox test). Additionally, there was no additive or synergistic effect observed when combining 1.CP1 with 1.SPP2, showing antibodies against CP1 have little to no impact in this model where mice were challenged with a relatively low dose of bacteria (2.1 x 10^4^ CFU). These results are consistent with previously reported works showing that anti-capsular antibodies and genetically inactivated toxoid vaccines can protect in lethal challenge models against a variety of serotypes (49). To mimic clinical observations where PCV13 immunization is not protective against ST-3, an adjustment to the immunization schedule and an increase in the challenge dose of bacteria was required.

**Figure 4:**
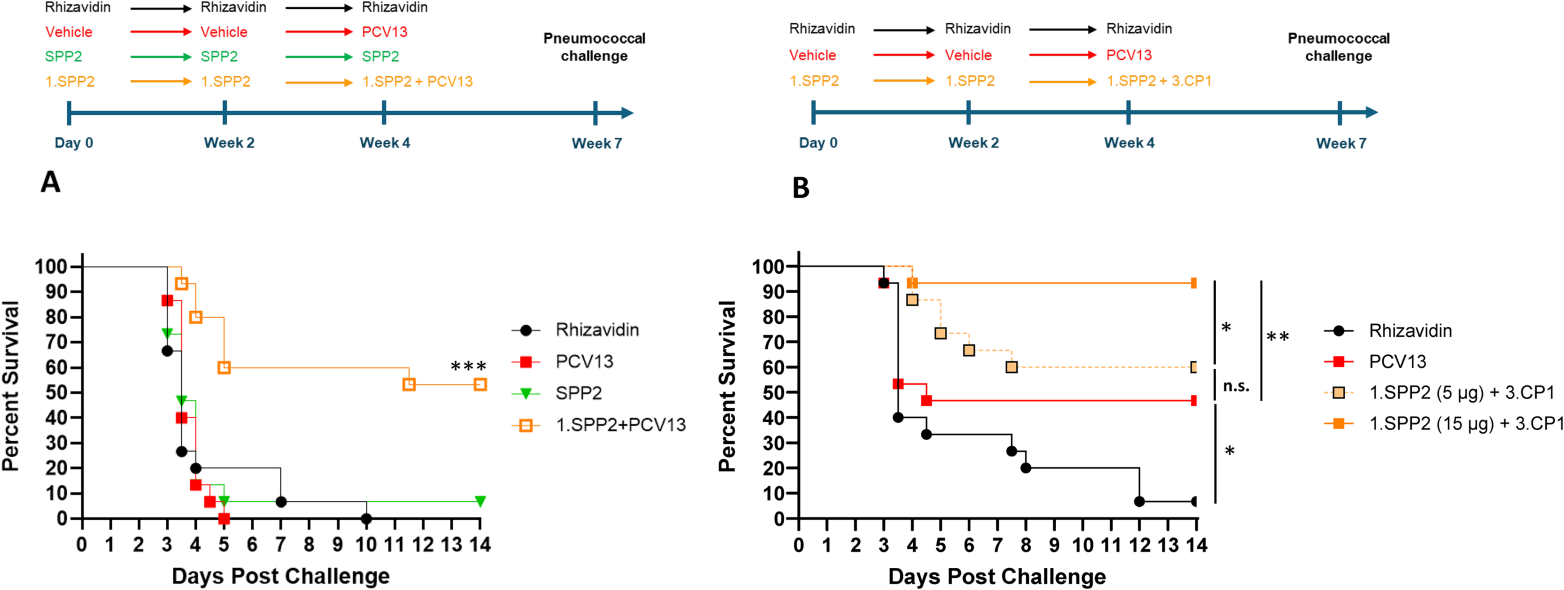
SPP2 and CPS3 protect synergistically against high dose sepsis challenge with ST-3. **(A)** Mice (n=15 for all groups) were immunized three times, two weeks apart with rhizavidin (5 μg/dose), SPP2 protein (15 μg SPP2/dose), or SPP2 in MAPS on CPS1 (1.SPP2,15 μg SPP2/dose). At Week 4, naive animals (having received vehicle twice) were primed with PCV13 (0.88 μg/PS/dose) and animals that received 1.SPP2 twice were given PCV13 (0.88 μg/PS/dose) in addition to 1.SPP2. At Week 7, mice were challenged intraperitoneally with 1.0 × 10^7^ CFU of S. pneumoniae strain AR003 and survival was monitored for up to two weeks post-challenge (***p<0.001 with Mantel-Cox test for 1.SPP2+PCV13 compared with the other groups). **(B)** Mice (n=15 for all groups) were immunized three times, two weeks apart with rhizavidin (5 μg/dose) or SPP2 in MAPS on CPS1 (1.SPP2, 15 μg SPP2/dose or 5 µg SPP2/dose). At week 4, naive animals (having received vehicle twice) were primed with PCV13 (0.88 μg/PS/dose) and animals that previously received 1.SPP2 were given CP1 in MAPS on CPS3 (3.CP1, 0.88 μg CPS3/dose) in addition to 1.SPP2. At Week 7, mice were challenged intraperitoneally with 6.7 × 10^6^ CFU of S. pneumoniae strain AR003. After challenge, all animals were monitored twice a day and euthanized at clinical endpoints. The percent survival of each group was plotted over two weeks post challenge and analyzed using a Mantel-Cox test for significance (*p<0.05, **p<0.01, ns = not significant).

Mice were completely protected by three injections of PCV13 or Pn-MAPS30plus at a vaccine dose as low as 0.04 µg/PS when challenged with 1.5 x 10^5^ CFU (**Supplementary Figure 2**). Interestingly, 1.SPP2 immunization alone was no longer protective when mice were challenged with this dose of bacteria, which is approximately 7-fold higher than the one used in **Figure 3**. Additionally, a single injection of PCV13, 3.CP1, or 3.CP1 + 1.SPP2 (0.88 µg/CPS3 dose) was also completely protective when mice were challenged with 1.5 x 10^5^ CFU (**Supplementary Figure 3**). A model calibration study was performed where mice were immunized with a single injection of PCV13 (0.88 µg/PS) and challenged with inoculum doses ranging from 3.5 x 10^5^ to 7.3 x 10^6^ CFU. The results of this model calibration study showed that a single injection of PCV13 required >6 x 10^6^ CFU challenge dose for protection to be meaningfully reduced (**Supplementary Figure 1A**). This high challenge dose was used for subsequent experiments to differentiate immunization with CPS3-only vaccines from those containing both CPS3 and SPP2. In **Figure 4A**, the synergistic protection of SPP2 and CPS3 is shown. Immunization with SPP2 or PCV13 alone was immunogenic (**Supplementary Figure 4**), but not protective at high challenge doses (1.0 × 10^7^ CFU). However, the combination of 1.SPP2 and PCV13 significantly protected the animals (p<0.001, Mantel-Cox). Additionally, **Figure 4B** shows a second experiment where animals were challenged with a slightly lower dose of bacteria resulting in partial protection in the PCV13 immunization group. The results also showed that by combining CP1 in a MAPS complex containing CPS3 (3.CP1) with 1.SPP2 at a 15 µg dose, 95% survival was achieved, which is significantly better than the PCV13-immunized group (p<0.01, Mantel-Cox). Additionally, the group immunized with 3.CP1 and 1.SPP2 at a 15 µg dose was significantly more protected than the group receiving 3.CP1 and 1.SPP2 at a 5 µg dose (p<0.05, Mantel-Cox), demonstrating a dose-response to 1.SPP2. Taken together, these results show that the protection offered by traditional glycoconjugates can be overcome with sufficiently high challenge doses of bacteria. Additionally, our results indicate that immunization with SPP2 and CPS3 glycocomplexes synergizes to confer protection in a high-dose model of type 3 pneumococcal sepsis.

### SPP2 immunogenicity in Pn-MAPS30plus

**Figure 5:**
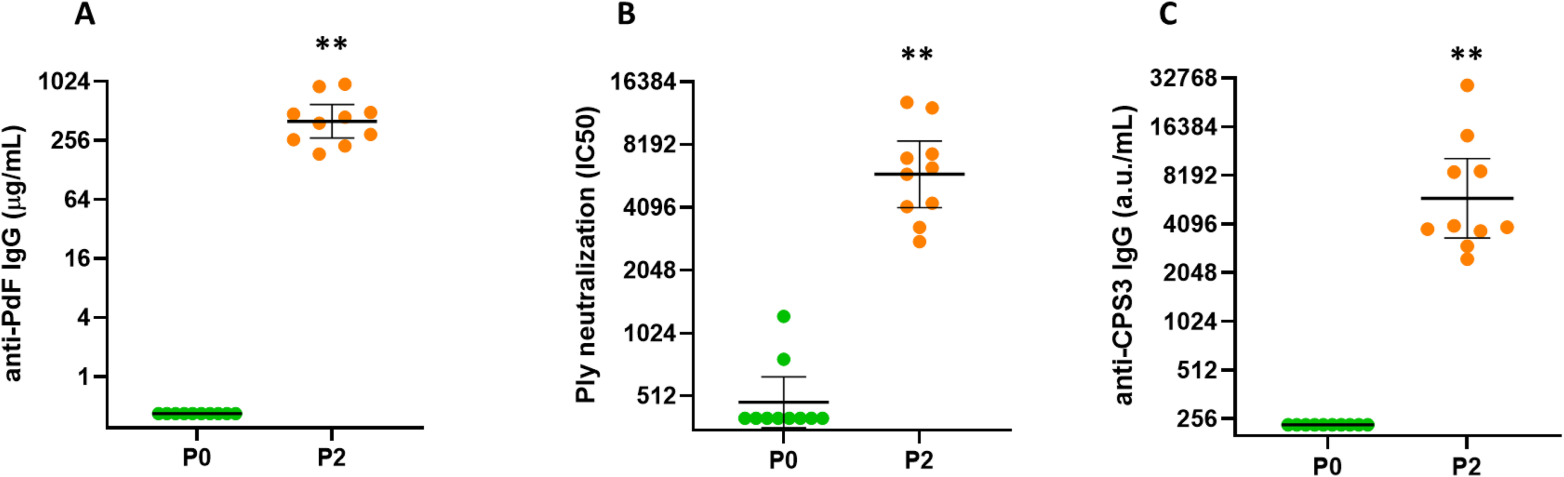
Immunogenicity of Pn-MAPS30plus. Rabbits (n=10 per group) were immunized intramuscularly at two occasions, two weeks apart with Pn-MAPS30plus, a vaccine candidate that contains CPS3 and other capsular polysaccharides from more than 30 serotypes combined with biotin-binding fusion proteins CP1 and SPP2 in MAPS complexes. P0 and P2 represent bleeds after 0 and 2 immunizations. **(A)** Anti-toxoid IgG concentrations, as determined by ELISA on the toxoid domain of SPP2 (PdF). Concentration values were determined by comparing samples to a common reference standard on each assay plate. The lower limit of quantification (LLOQ) was 0.32 µg/mL **(B)** Ply neutralization titers. Serum samples were diluted and pre-incubated with Ply prior to running a hemolysis assay. Percent inhibition of hemolysis vs serum dilution was plotted and fit to a 4PL curve to determine the serum concentration required to achieve 50% inhibition of hemolysis (IC50). If a sample did not achieve 50% inhibition, its IC50 is reported as the lowest serum dilution tested, which was 1/400. **(C)** Anti-CPS3 IgG measured by electrochemiluminescence. Biotinylated CPS3 was coated on plates, blocked, and incubated with rabbit serum samples at various dilutions. Arbitrary units (a.u./mL) of samples were determined by comparing samples to a common reference standard on each assay plate. The LLOQ for this assay was 235 a.u./mL. In all three graphs, the horizontal bars are the geometric mean concentrations of the groups and error bars are 95% confidence intervals. Each P0 and P2 timepoint was compared using Wilcoxon matched-pairs signed rank test and were significantly different (**p=0.002).

A vaccine candidate that contains CPS3 and other capsular pneumococcal polysaccharides from more than 30 serotypes combined with biotin-binding fusion proteins CP1 and SPP2 in complexes (Pn-MAPS30plus) was evaluated for immunogenicity in rabbits. Pn-MAPS30plus generated a robust immune response to both SPP2 and CPS3, as measured by anti-PdF IgG concentrations and Ply neutralization titers and anti-CPS3 IgG concentrations, respectively (**Figure 5**).

### Passive immunization with anti-SPP2 and anti-CPS3 provide synergistic protection

**Figure 6:**
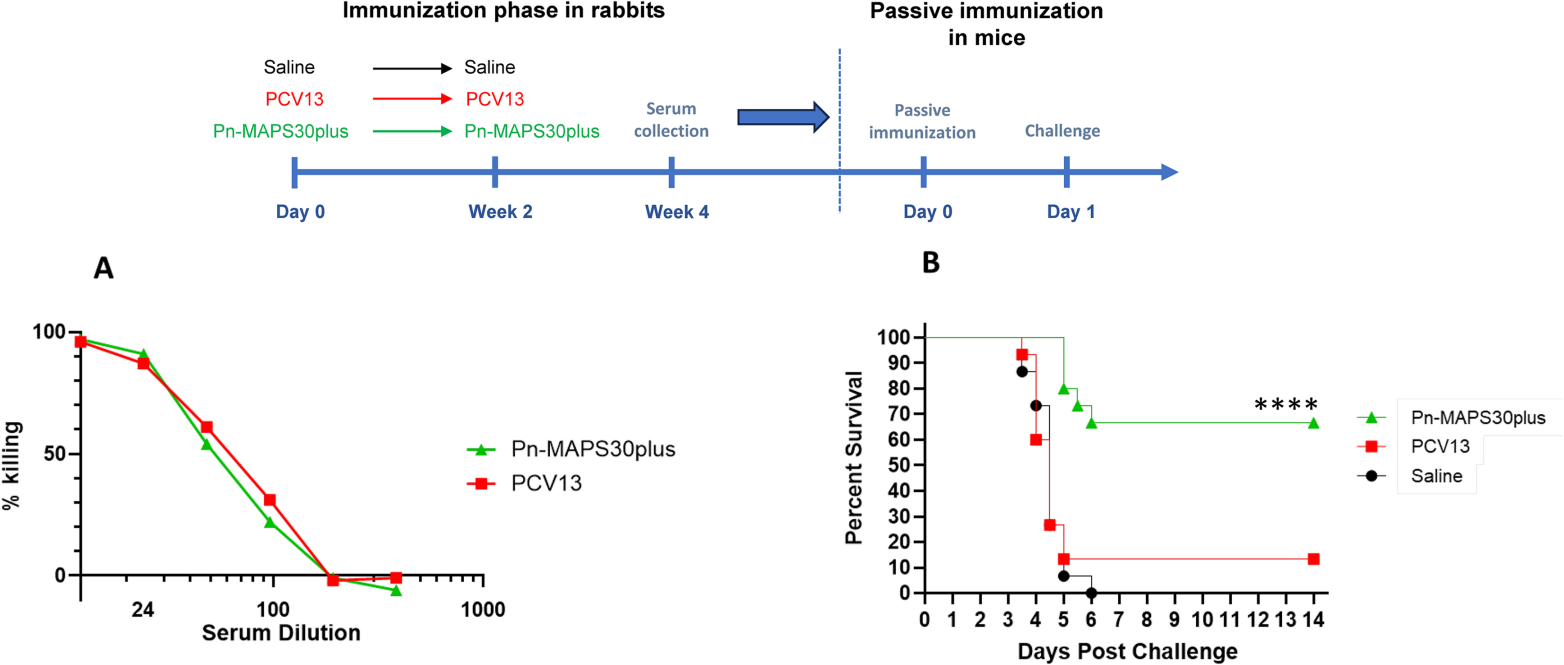
Passive Immunization with anti-SPP2 and anti-CPS3 provide synergistic protection. One rabbit serum from the Pn-MAPS30plus group was selected for the passive immunization experiment. A pool of anti-PCV13 sera was created to have an anti-CPS3 IgG titer matching that of the selected MAPS-immunized rabbit serum, which was confirmed by measuring their respective killing activity against Type 3 Pneumococcus challenge strain **(A)**. To that purpose, bacteria were incubated with diluted serum or assay buffer prior to incubation with baby rabbit complement and differentiated HL60s. Percent killing was normalized by the no serum control. **(B)** Percent survival of mice passively immunized with either the Pn-MAPS30plus rabbit serum or the anti-PCV13 rabbit serum pool and challenged with a high dose sepsis model. Mice received 200 μL of serum or saline via intraperitoneal injection one day prior to an intraperitoneal challenge with 3.8 × 10^4^ CFU of Type 3 pneumococcus. Mice were monitored twice a day and euthanized at clinical endpoints. The percent survival of each group was plotted over two weeks post challenge. Survival curves were compared using the Mantel-Cox test. Only the group receiving Pn-MAPS30plus antisera was significantly protected vs saline group (p<0.0001). Additionally, the Pn-MAPS30plus group was significantly protected against the PCV13 group (****p=0.0001).

We selected a serum sample from the Pn-MAPS30plus group based on anti-CPS3 IgG and Ply IC50 titers. The sample is in the upper quartile of Ply IC50 for the group and had the highest anti-CPS3 IgG value. A pool of PCV13 P2 sera was created to have an anti-CPS3 IgG titer matched to the selected Pn-MAPS30plus-immunized rabbit serum. An OPA was used to measure the functional titers of these two samples against AR003. **Figure 6A** shows that PCV13 and Pn-MAPS30plus sera had nearly identical killing activities, indicating that they were matched for anti-CPS3 activity. A model calibration study was performed where mice were passively immunized with the PCV13-immunized serum pool and challenged with inoculum doses ranging from 1.3 x 10^3^ to 2.2 x 10^4^ CFU. The results of this model calibration study showed that for a serum sample with this anti-CPS3 functional titer, a challenge dose >2 x 10^4^ CFU was required to differentiate between antisera generated by immunization with CPS3 alone from those containing both CPS3 and SPP2 (**Supplementary Figure 1B**). **Figure 6B** shows significant protection in mice that received the Pn-MAPS30plus sera (p<0.001 when compared with saline, Mantel-Cox) and no protection in the mice that received the PCV13 serum (p=0.72 vs saline, Mantel-Cox).

## Discussion

The study highlights the limitations of current PCVs, particularly the inability of PCV13 to protect against type 3 IPD (18, 50–53). This limitation may stem from the unique properties of ST-3. One of these properties include its dense capsular polysaccharide that is readily shed due to the absence of covalent attachment to the bacterial cell wall (27, 54), with as consequence the absorption of anti-CPS3 antibodies by shed CPS3 targets. Further, the type 3 component of PCV13 may simply exhibit insufficient immunogenicity (25). These observations underscore the need for vaccines with enhanced immunogenicity to CPS3 or novel approaches, such as combining polysaccharides with protein virulence factors that are conserved across pneumococci.

Immunization with a genetically inactivated Ply toxoid in the form of a rhizavidin fusion protein (SPP2) demonstrated protective efficacy against a lethal challenge with ST-3 in mice, under conditions where traditional glycoconjugates are completely protective. This showed that the immune response generated by SPP2 alone can contribute to protection. To model earlier clinical observations of PCV13 poor effectiveness against ST-3- caused IPD, we developed a high-dose challenge model in which PCV13 was not highly protective. In this high-dose challenge model, protection was observed when SPP2 and CPS3 were combined in active immunization, suggesting a synergy between these two protective mechanisms of action. In a prior study, Thanawastien et al (55), showed the protective efficacy of combining a genetically inactivated toxoid with PCV13, when neither PCV13 nor the toxoid alone was protective. However, as the authors note, differences in anti-type 6B antibody concentrations in the two groups that received PCV13 may have confounded the results. Here, we selected sera with comparable anti-type 3 antibody concentrations and OPKA activities, which strongly support a vaccine approach that includes both pneumococcal polysaccharides and conserved pneumococcal virulence proteins to overcome the resistance of ST-3 pneumococcal disease to traditional glycoconjugate vaccines.

The Pn-MAPS30plus vaccine candidate evaluated in this study complexes over 30 pneumococcal polysaccharides, including CPS3, with CP1 and SPP2 fusion proteins. Immunization with Pn-MAPS30plus elicited a robust opsonophagocytic response to CPS3 and Ply-neutralizing antibodies in rabbits. Passive immunization of mice with antiserum generated by Pn-MAPS30plus conferred significant protection against death. In stark contrast, anti-CPS3 IgG concentration-matched serum derived from PCV13 immunization failed to protect mice challenged with ST-3 at a dose specifically selected to differentiate between these sera samples. These findings confirmed a synergistic interaction between the protein- and PS-directed antibodies generated by Pn-MAPS30plus immunization.

Despite these promising results, several limitations of this study must be acknowledged. Animal models, while invaluable for preliminary investigations, may not fully replicate the human immune response to vaccination. Further, this study did not explore the impact of anti-CPS3 and anti-protein immune responses in the context of a pneumococcal pneumonia. Therefore, the protective efficacy observed in this study requires further validation in preclinical models, as well as in human clinical trials. Recent efforts to develop an experimental human pneumococcal challenge for ST-3 provide an opportunity to directly assess the efficacy of existing PCVs and novel vaccine candidates containing conserved pneumococcal proteins against ST-3 colonization in a clinical setting (56, 57).

In conclusion, the findings presented in this study strongly advocate for the development of vaccines that combine pneumococcal polysaccharides with conserved protein virulence factors, including Ply toxoids. Such vaccines have the potential to address the current gap in the current pneumococcal vaccination strategy by offering enhanced protection against ST-3 IPD.

## Data Availability

The datasets presented in this article are not openly available due to company restrictions but are available from the corresponding author upon reasonable request. Data are located in controlled access data storage at GSK. There are restrictions to the availability of MAPS and related items in this work because of multiple company-owned patent related to these products. Requests to access the datasets should be directed to taylor.c.stevenson@gsk.com.
