# Supplementary material for: Antibodies against polysaccharide type 3 and pneumococcal proteins demonstrate synergistic protective effect in a highly virulent type 3 invasive disease model in mice
Source: Front Immunol. 2025 Dec 12;16:1707686. doi: 10.3389/fimmu.2025.1707686 (PMC12741848; doi:10.3389/fimmu.2025.1707686)
Supplement: Supplementary file 1 [file DataSheet1.pdf]

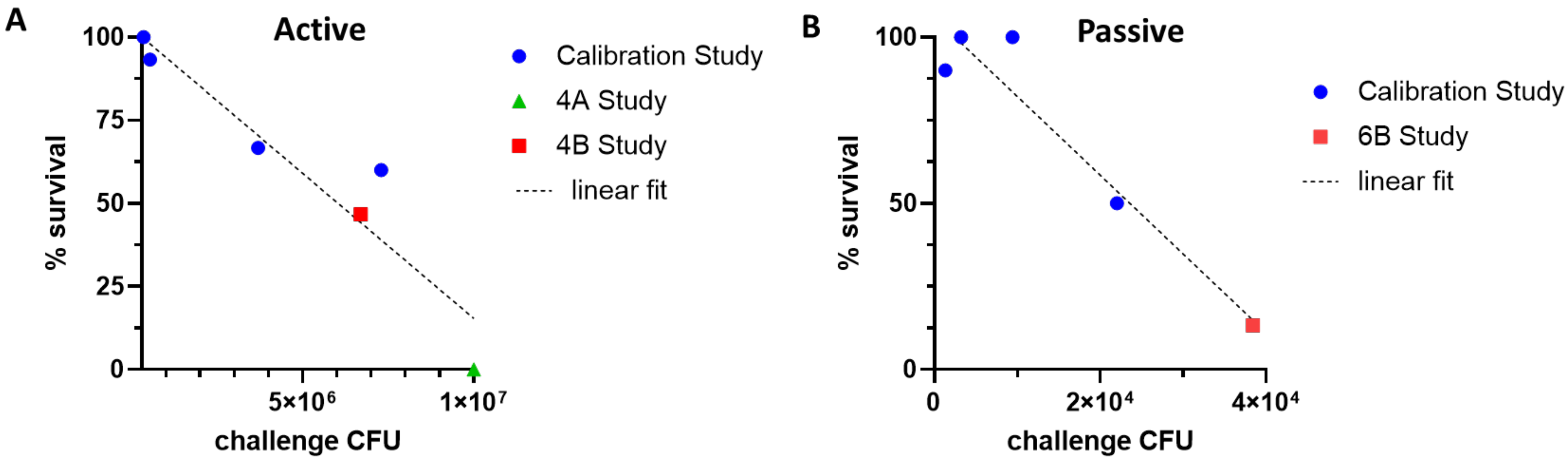

Supplementary Figure 1. A. Percent survival of mice actively immunized with PCV13 (0.88 µg/PS) once three weeks prior to challenge with various CFU of ST-3. Mice received 200 µL of serum via intraperitoneal injection one day prior to an intraperitoneal challenge with Type 3 pneumococcus. Mice were monitored twice a day and euthanized at clinical endpoints. The percent survival of each group at the end of two weeks post challenge is reported. Data shown are a composite of three independent studies (Calibration Study, Figure 4A study, and Figure 4B study, n = 15 mice/group). Data were fit to a simple linear model to aid with visualization of dose-response. **B.** Percent survival of mice passively immunized with anti-PCV13 rabbit serum pool and challenged with various CFU of ST-3. Mice received 200 µL of serum via intraperitoneal injection one day prior to an intraperitoneal challenge with Type 3 pneumococcus. Mice were monitored twice a day and euthanized at clinical endpoints. The percent survival of each group at the end of two weeks post challenge is reported. Data shown are a composite of two independent studies (Calibration Study n = 10 mice/group, Figure 6B Study n = 15/group). Data were fit to a simple linear model to aid with visualization of dose-response.
