# Supplementary material for: Antibodies against polysaccharide type 3 and pneumococcal proteins demonstrate synergistic protective effect in a highly virulent type 3 invasive disease model in mice
Source: Front Immunol. 2025 Dec 12;16:1707686. doi: 10.3389/fimmu.2025.1707686 (PMC12741848; doi:10.3389/fimmu.2025.1707686)
Supplement: Supplementary file 3 [file DataSheet3.pdf]

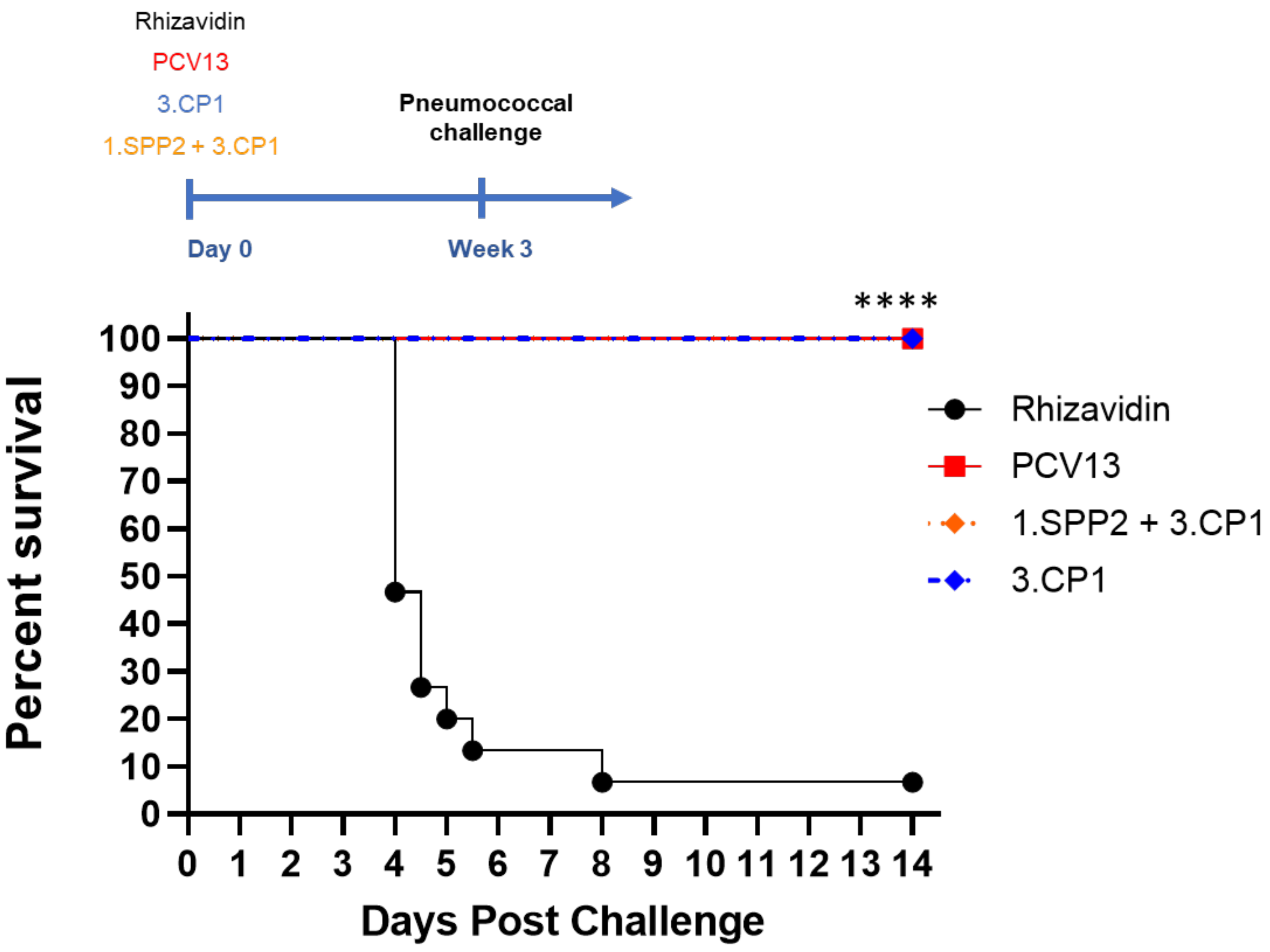

Supplementary Figure 3. PCV13 and 3.CP1 protect against sepsis challenge with ST-3. Mice (n=15 for each group) were immunized once with rhizavidin (5 µg/dose), PCV13 (0.88 µg/PS/dose), CP1 in MAPS on CPS3 (3.CP1,0.88 µg CPS3/dose), or 3.CP1 (0.88 µg CPS3 /dose) and SPP2 in MAPS on CPS1 (1.SPP2, 15 µg SPP2/dose). Three weeks after immunization, mice were challenged with 1.5×10^5 CFU of *S. pneumoniae* strain AR003 via intraperitoneal injection. Mice were monitored twice a day and euthanized at clinical endpoints. The percent survival of each group was plotted over two weeks post challenge. All groups showed significant protection (****, p<0.0001 with Mantel-Cox test) compared with rhizavidin-immunized control group.
