# Supplementary material for: Antibodies against polysaccharide type 3 and pneumococcal proteins demonstrate synergistic protective effect in a highly virulent type 3 invasive disease model in mice
Source: Front Immunol. 2025 Dec 12;16:1707686. doi: 10.3389/fimmu.2025.1707686 (PMC12741848; doi:10.3389/fimmu.2025.1707686)
Supplement: Supplementary file 4 [file DataSheet4.pdf]

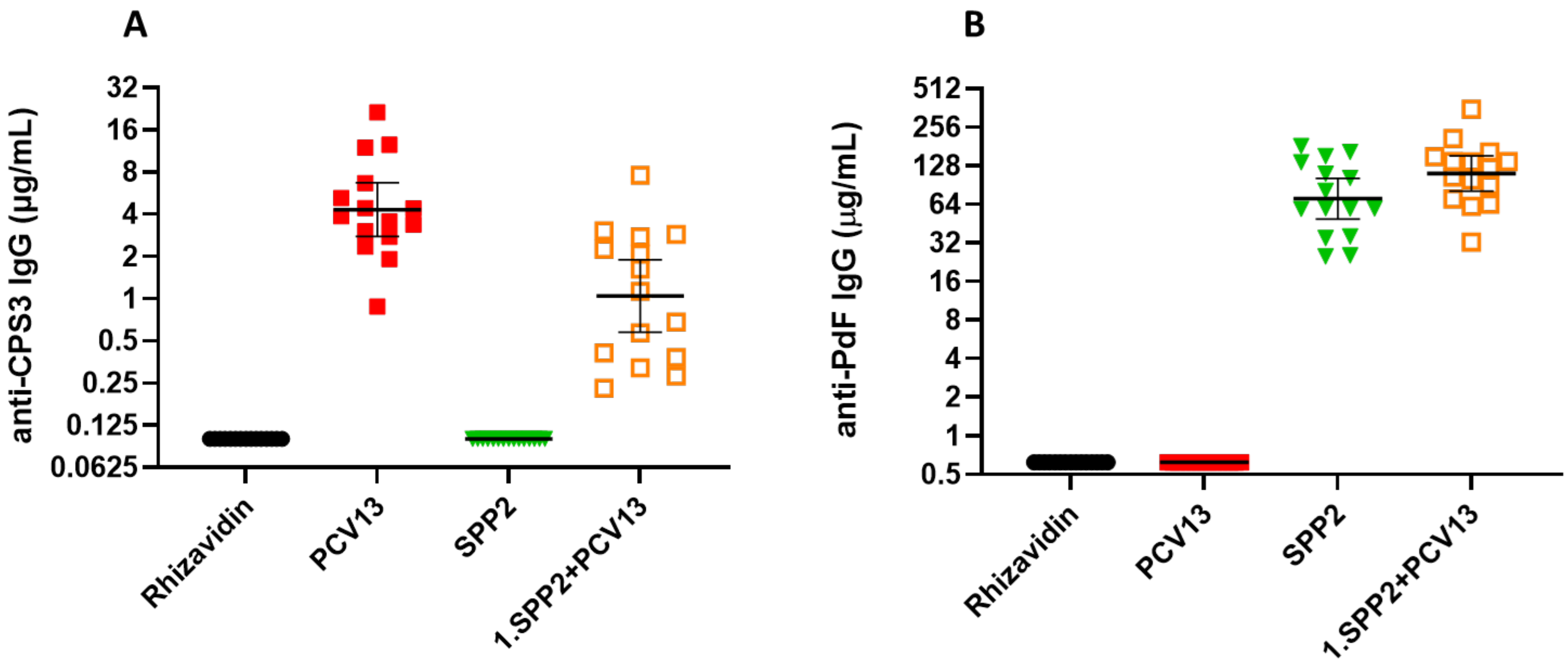

Supplementary Figure 4. Anti-CPS3 and anti-PdF IgG concentrations in mouse active immunization. Antigen-specific IgG concentration in mice (n=15 per group) two weeks post third immunization from the challenge experiment with survival results in Figure 4A. **A.** Anti-CPS3 IgG was measured by electrochemiluminescence. Biotinylated CPS3 was coated on plates, blocked, and incubated with mouse serum samples at various dilutions. Concentration values were determined by comparing samples to a common reference standard on each assay plate. The LLOQ for this assay was 0.1 µg/mL. **B.** Anti-PdF IgG concentrations, as determined by ELISA on the toxoid domain of SPP2 (PdF) and incubated with mouse serum samples at various dilutions. Concentration values were determined by comparing samples to a common reference standard on each assay plate. The lower limit of quantification (LLOQ) was 0.63 µg/mL. In both graphs, the horizontal bars are the geometric mean concentrations of the groups and error bars are 95% confidence intervals.
